# Elevated *CCL19*/*CCR7* Expression During the Disease Process of Primary Sjögren's Syndrome

**DOI:** 10.3389/fimmu.2019.00795

**Published:** 2019-04-24

**Authors:** Zhenwei Liu, Fengxia Li, Axiao Pan, Huangqi Xue, Shan Jiang, Chengwei Zhu, Mengmeng Jin, Jinxia Fang, Xiaochun Zhu, Matthew A. Brown, Xiaobing Wang

**Affiliations:** ^1^Institute of Genomic Medicine, Wenzhou Medical University, Wenzhou, China; ^2^Rheumatology Department, The First Affiliated Hospital of Wenzhou Medical University, Wenzhou, China; ^3^Institute of Health and Biomedical Innovation, School of Biomedical Sciences, Queensland University of Technology (QUT) at Translational Research Institute, Brisbane, QLD, Australia; ^4^Centre for Precision Medicine, The First Affiliated Hospital of Wenzhou Medical University, Wenzhou, China

**Keywords:** Primary Sjögren's syndrome, RNA-sequencing, gene expression, immunological synapse, *CCL19*/*CCR7*

## Abstract

Primary Sjögren's syndrome (pSS) is a common chronic autoimmune disease characterized by a high prevalence of autoantibodies and lymphocyte-mediated exocrine gland damage. To enhance our understanding of the mechanisms underlying the progression of the disease and to discover potential biomarkers for the early diagnosis of pSS, we applied RNA sequencing to compare the gene expression patterns in minor salivary glands between pSS patients and non-pSS. A total of 293 differentially expressed genes (DEGs) were detected in pSS vs. non-pSS (FDR < 0.05, fold changes > 2). Of these DEGs, 285 (97.26%) were up-regulated, with most being involved in immune system activation, especially in the formation of the immunological synapse. Significantly elevated *CCL19*/*CCR7* expression in the salivary gland was found to be related to anti-Sjögren's syndrome-related antigen A (SSA) antibody and IgG levels in pSS patients, which was further confirmed in a larger cohort. Up-regulated gene expression showed strong discriminatory accuracy in identifying pSS with area under the curve of 0.98 using receiver operating characteristic curve analysis. In conclusion, gene expression changes in pSS include strong markers of immunological activation and have good discriminatory power in identifying patients with pSS.

## Introduction

Primary Sjögren's syndrome (pSS) is a chronic, systemic autoimmune disease characterized by a high prevalence of typical autoantibodies Ro/Sjögren's syndrome-related antigen A (SSA) and La/Sjögren's syndrome-related antigen B (SSB), and lymphocytic-mediated exocrine glands damage (1–3). The disease affects 0.2 to 1.2% of the population, particularly affecting middle-aged women (4), with half of patients having the primary form. The clinical presentation of pSS is extremely heterogeneous, varying from mild disease with just sicca symptoms to a severe systemic disease involving multiple systems (4–6). Systemic disease is associated with a poor prognosis and increased mortality, and for which there is a great unmet therapeutic need (7). There is also a great need for improved diagnostic markers, as on average it takes 7 years for pSS patients from onset of symptoms to final diagnosis (8).

Identification of biomarkers for pSS may also facilitate dissection of the molecular mechanisms underlying the progression of pSS, and potentially assist with improving on current classification criteria of pSS cases (9–11). An integrative multiomic investigation of human whole-blood has found that SS gene signatures are overlapped with SS-causing genes, and that expression trait loci analyses and cytotoxic CD8^+^ T cells are associated with SS pathology (12). In addition, interferon (IFN)-inducible genes or other genes associated with inflammatory and immune-related pathways are often overexpressed in the peripheral blood of pSS cases, implying the involvement of innate and adaptive immune processes in the pathogenesis of pSS (13). Furthermore, gene expression profiling of minor salivary glands (MSGs) revealed a distinct gene expression signature in pSS patients compared to healthy controls and identified groups of genes that are up-regulated (such as type I interferon, *CXCL13*, major histocompatibility complex genes and lymphocyte activation factors) or down-regulated (such as Bcl-2) during disease progress (14). Moreover, IFN pathways have also been shown to be activated in pSS compared with normal controls, including up-regulation of two Toll-like receptors (*TLR8* and *TLR9*) and recruitment of plasmacytoid dendritic cells (pDCs) in the target organs in compared with normal control (15). In pSS, observations of up-regulation of both endosomal *TLR7* and its downstream signaling molecules in IFN-positive pDCs and monocytes have further confirmed the involvement of pathways underlying IFN type I bioactivity and pDCs, while IFN-negative patients had a contrasting expression (16). These findings support pSS being the result of pathogenic interaction between the innate and adaptive immune system, and environmental factors in the pathogenesis of pSS (15, 16).

However, all these previous studies have investigated differences in genomic variants or gene expression between pSS cases and heathy individuals. In this study, we first applied RNA sequencing to compare gene expressions in MSGs from established pSS patients and non-pSS, who have typical clinical symptoms but do not yet meet the diagnostic criteria. Investigation of MSG gene expression profiles in pSS will enhance our understanding of the mechanisms underlying the progression of the disease.

## Materials and Methods

### Patients and Sample Preparation

Fifteen patients with pSS together with 12 non-pSS subjects for RNA sequencing were recruited from the First Affiliated Hospital of Wenzhou Medical University, China. All pSS patients fulfilled the 2016 American College of Rheumatology (ACR) /European League Against Rheumatism (EULAR) classification criteria (11) or 2012 ACR classification criteria (10) for pSS. The non-pSS were subjects who had experienced subjective symptoms of dryness, but do not meet the classification criteria for pSS. The clinical features of pSS patients and non-pSS were assessed by high IgG (IgG ≥ 16 g/L), focus score, anti-SSA positivity, anti-SSB positivity, antinuclear antibody (ANA) and whole unstimulated saliva flow, all of which were summarized in Table 1. A further 118 additional pSS subjects and 118 non-pSS were recruited for further validation of candidate gene expression from the First Affiliated Hospital of Wenzhou Medical University, China (Supplementary Table 1). A labial gland biopsy was performed at the time of the baseline evaluation on each subject, in which salivary glands were obtained from the inner surface of the lower lip under local anesthesia. These biopsy samples were then processed by the local pathology departments using the approach of paraffin embedding, sectioning, and hematoxylin and eosin staining. Histopathological analysis was performed by 2 experienced pathologists that diagnosed the focal lymphocytic sialadenitis based on a focal score of one or more lymphocytic foci (>50 lymphocytes/4 mm^2^) (9). Biopsy samples were snap-frozen and kept in liquid nitrogen until RNA extraction.

**Table 1:** The clinical characteristics of the pSS patients and non-pSS subjects.

**Sample**	**Group**	**Age**	**SSA**	**SSB**	**Focus score**	**ANA Titers (Pattern)**	**High IgG**	**Whole unstimulated Saliva flow (ml/15 min)**
N1608171	Non-pSS	56	–	–	0	Negative	0	1
N1609261	Non-pSS	67	+	+	0	1:100 (nuclear speckled)	0	1.4
N1609281	Non-pSS	37	+	–	0	1:100 (nuclear speckled)	0	0.8
N1609291	Non-pSS	55	+	–	0	1:100 (nuclear speckled)	0	1.3
N1611082	Non-pSS	34	–	–	1	Negative	0	1.2
N1612131	Non-pSS	65	–	–	0	1:100 (cytoplasmic reticular)	0	1.4
N1701063	Non-pSS	40	+	–	0	1:100 (nuclear speckled)	0	1.3
N1701161	Non-pSS	60	–	–	0	Negative	0	1.2
N1701241	Non-pSS	30	+	+	0	1:100 (nuclear speckled)	1	0.7
N1701243	Non-pSS	55	–	–	0	1:100(nuclear homogeneous)	0	0.5
N1703281	Non-pSS	40	–	–	0	Negative	1	1.1
N1709057	Non-pSS	50	–	–	0	1:100 (cytoplasmic speckled)	0	1.2
Y1610111	pSS	43	+	+	2	1:320 (nuclear speckled)	1	0.4
Y1610201	pSS	63	+	+	1	1:100 (nuclear speckled)	1	0.6
Y1610271	pSS	69	+	+	2	1:100 (nuclear speckled)	0	0.5
Y1611101	pSS	58	+	+	1	1:100 (nuclear speckled)	1	0.7
Y1612021	pSS	29	+	+	3	1:3200 (nuclear speckled)	1	0.9
Y1612091	pSS	57	+	+	2	1:320 (nuclear speckled)	1	1.2
Y1612231	pSS	27	+	–	2	1:100 (nuclear speckled)	1	0.7
Y1702101	pSS	48	–	–	1	1:1000 (centromere)	0	0.4
Y1702211	pSS	45	–	–	2	1:320 (nuclear homogeneous)	0	1.3
Y1703072	pSS	56	+	+	1	1:3200 (nuclear speckled)	1	1.1
Y1704061	pSS	57	+	+	1	1:100 (nuclear speckled)	1	1.2
Y1704141	pSS	68	+	+	3	1:1000 (nuclear speckled)	1	1.4
Y1704182	pSS	23	+	+	2	1:320 (nuclear speckled)	0	1.2
Y1704281	pSS	33	+	+	1	1:100 (nuclear speckled)	0	0.7
Y1705161	pSS	59	–	+	3	1:1000 (nuclear homogeneous)	1	0.7

*The number of “1” in “High IgG” group represents the IgG level ≥16 g/L, while “0” means 7 g/L < IgG < 16 g/L. Subjects with focus score ≥1 indicate the positivity for histopathology*.

### Detection of Autoantibodies and Antinuclear Antibody

The assessments of anti-SSA and anti-SSB were performed using immunoblotting commercial kits (EUROIMMUN) by automated EUROBlotmaster platform (Euroimmun AG, Lübeck, Germany) in the central clinical laboratory. The result was evaluated using the EUROLineScan software (Euroimmun AG, Lübeck, Germany). Compared with quality control stripe, a white stripe is considered to be negative and colored strip is identified as positive. Both anti-SSA and anti-SSB have specific location in the membrane.

ANA was detected by indirect immunofluorescence (IIF) commercial kit (EUROIMMUN). The final result was checked manually under fluorescence microscope. If no fluorescence is observed, it should be considered as negative. The titer is the highest dilution ratio for ANA that keep positive compared with the negative serum control.

### RNA Extraction, cDNA Library Preparation and Sequencing

Frozen labial glands were processed for total RNA isolation using TRIzol® Reagent (Invitrogen), as described in the manufacturer's protocols. RNA purity was examined with a Nano Photometer spectrophotometer (Implen, CA, USA). The quality of RNA used for cDNA library preparations was verified using an RNA 6000 Nano kit with a Bioanalyzer 2100 system (Agilent Technologies, CA, USA).

Three micrograms of RNA per sample was used as input material for sequencing library generation using an NEBNext Ultra™ RNA Library Prep Kit for Illumina (NEB, USA) following the manufacturer's recommendations. Index codes were added to label the sequences for each sample. Briefly, mRNA was purified from total RNA using poly-T oligo-attached magnetic beads. Fragmentation was carried out using divalent cations under elevated temperature in NEBNext First Strand Synthesis Reaction Buffer (5X). First strand cDNA was synthesized using random hexamer primers and M-MuLV Reverse Transcriptase (RNase H-). Second strand cDNA synthesis was subsequently performed using DNA polymerase I and RNase H. Remaining overhangs were converted into blunt ends via exonuclease/polymerase activity. The prepared libraries were sequenced on an Illumina HiSeq platform.

### Data Preprocessing

To evaluate read quality, we filtered low-quality reads using Trim Galore and trimmed adapters using Cutadapt as described in a previous study (17). FastQC assessment reports of sequence reads were obtained before and after preprocessing. All subsequent analyses were conducted using clean reads. The sequencing data were aligned using a RefSeq hg19 reference with STAR software.

### Identification of Differentially Expressed Genes

We used the DEseq2 (18) package to normalize the RNA-seq data and performed a two-dimensional principal component analysis (PCA) and hierarchical clustering to visualize the similarities and differences between the pSS tissues and non-pSS tissues. Subsequently, differential gene expression analysis was performed using DEseq2. A gene was considered a differentially expressed gene (DEG) if it met the following criteria: a false discovery rate (FDR) <0.05 and a log_2_ fold Change (FC) < −1 or log_2_ FC > 1 in DEseq2. A volcano plot visualizing all DEGs between different subjects was constructed in R with the “ggplot2” package, and a heatmap for the DEGs was drawn using the R software package “pheatmap”.

There were 22 DEGs that were validated by quantitative real-time PCR (qPCR) to verify the robustness of RNA-seq. The correlation between RNA-seq and qPCR of the candidate genes was calculated by Pearson's test.

To investigate the effect of sample size on the numbers of detected DEGs, we randomly selected five to fifteen samples from pSS patients and non-pSS subjects, respectively, and performed 100 permutations. Then, differential gene expression analysis was performed as described previously.

### Quantitative Real-Time PCR Analysis

Total RNA was isolated using TRIzol® Reagent (Invitrogen) following the manufacturer's instructions, and 1 μg of total RNA was used for reverse transcription and qPCR with a GoTaq® 2-Step RT-qPCR System (Promega). A total of 15 DEGs were assayed by qPCR on an Applied Biosystems QuantStudio™ 3 Real-time PCR Instrument (ABI). After enzyme activation at 95°C for 2 min, 40 amplification cycles of 95°C for 15 s and 60°C for 60 s were performed. We repeated qPCR for at least three times. The relative log_2_ FCs in pSS samples compared with non-pSS samples were calculated according to the ^ΔΔ^Ct method.

### Gene Ontology and Kyoto Encyclopedia of Genes and Genomes Analysis of DEGs

The “ClueGO” plugin with integration of Gene Ontology (GO) terms in Cytoscape was used to construct separate biological process networks with the up-regulated genes and down-regulated genes. Functions associated with groups were partitioned based on significant functional associations between terms and gene sets. The Bonferroni step-down method was used for multiple correction with a threshold of 0.05 for corrected *P*-values.

Kyoto Encyclopedia of Genes and Genomes (KEGG) analysis of DEGs was performed the using ClusterProfiler (19) R package with the “enrichKEGG” function. Pathways with corrected *P* < 0.05 were considered significantly enriched for DEGs.

### Protein-Protein Interaction Network Analysis

Protein–protein interaction (PPI) data downloaded from the STRING v10 (20) database were used to create networks. A total of 100,000 permutations of genes and connections were evaluated to verify that the PPI networks were not random. Then, the PPI networks were visualized by using Cytoscape software (21).

### The Expression Changers of Both *CCL19* and *CCR7* in pSS Based on Clinical Indexes

We validated gene expression of *CCL19* and *CCR7* using qPCR in additional 118 pSS patients and 118 non-pSS for verifying the role of immunological synapse in the pSS. The correlations between expression of *CCL19* and *CCR7* in pSS and non-pSS were calculated by Pearson's test. We compared the differences in gene expression of *CCL19* and *CCR7* between 118 pSS patients and 118 non-pSS using two sample *t*-test. Then 118 pSS patients were divided into anti-SSA positive and negative groups or high IgG (IgG ≥16 g/L) and normal IgG (7 g/L < IgG < 16 g/L) groups, in which the significance test was performed with *t*-test. The same test was done in the 118 non-pSS.

### Phenotype Analysis of DEGs

We downloaded phenotype data from the Human Phenotype Ontology (HPO) database (22), and each phenotype corresponded to its associated genes. The Genes of each phenotype were intersected with DEGs, and phenotypic enrichment was obtained with a hypergeometric test.

### The Indexes for the Prediction of pSS Using Random Forest Model

Random Forest was applied to predict pSS based on up-regulated gene expression and other clinical indexes, including pathology, anti-SSA positivity, anti-SSB positivity and hypergammaglobulinemia. We implemented Random Forest using R package “randomForest,” in which 100 runs of cross-validation were performed. In each run, the dataset was randomly split into 70% and 30%, among which 70% of the samples were selected as train set to train the prediction model and the remaining were used to test the quality and reliability of the prediction model. ROC curve was performed using R package “ROCR” based on the 100 runs of cross-validation. Mean decrease accuracy and mean decrease Gini in the 100 runs were presented using “ggplot2” R package. ANOVA test was performed to analyze difference between the five indexes, multiple comparisons were using Nemenyi test.

## Results

### Prevalence of Up-Regulated Genes in the Development of pSS

**Figure 1:**
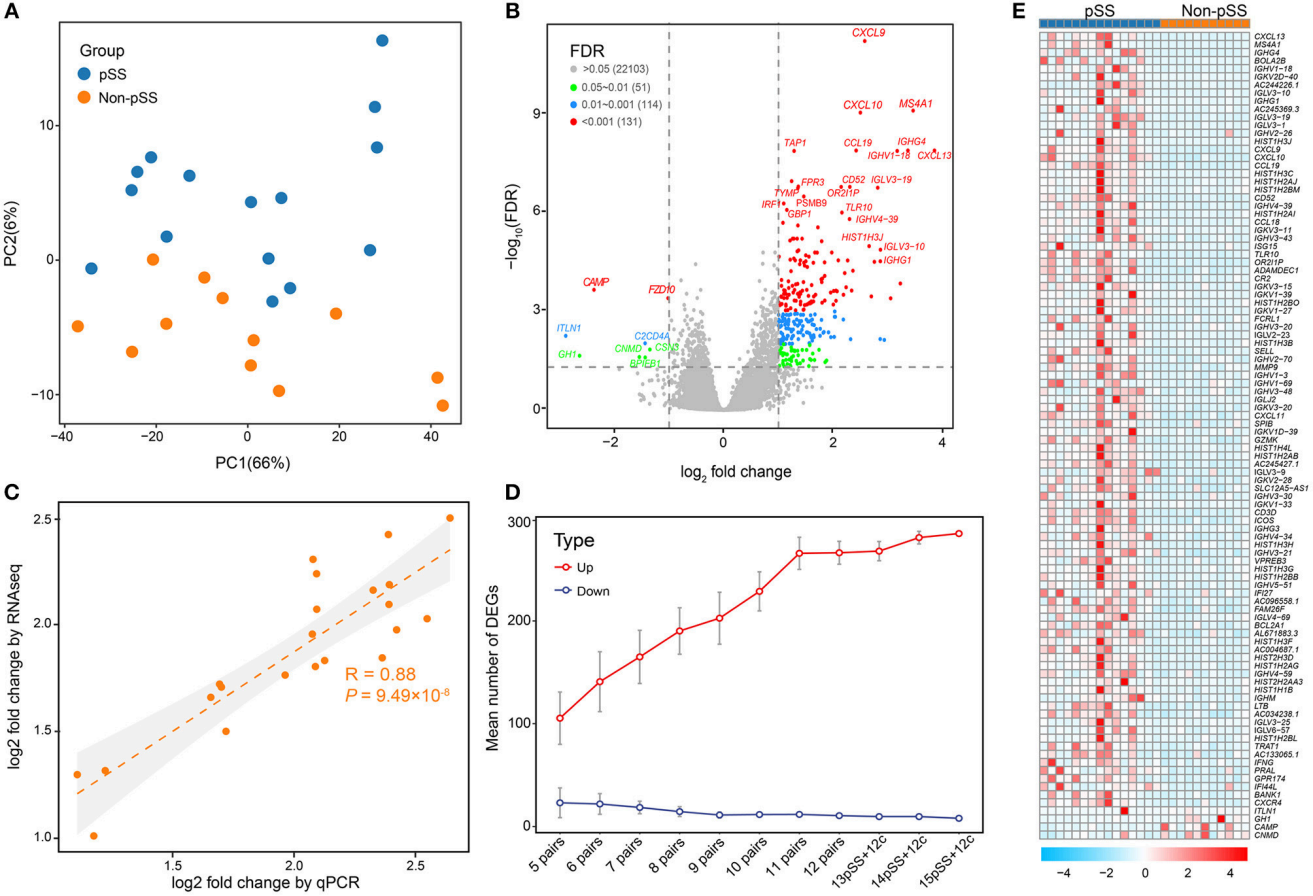
Comprehensive transcriptome analysis revealed massive gene up-regulation in the development of pSS**. (A)** PCA illustrating the individual differences in the RNA-seq expression profiles between the pSS and non-pSS groups for 27 samples. The red and blue circles represent the case and non-pSS groups, respectively. **(B)** Volcano plot highlighting DEGs in pSS. The red dots illustrate the 131 up-regulated or down-regulated DEGs (adjusted *P* < 0.001 and log_2_ FCs >1). The blue and green dots show, respectively, 114 DEGs with adjusted *P* < 0.01 and 51 DEGs with adjusted *P* < 0.05. **(C)** Correlations between RNA-seq and qPCR for the log_2_ FCs of DEGs, as analyzed by the Pearson's test (*R* = 0.88, *P* = 9.49 × 10^−8^). **(D)** Saturation curve showing the means and confidence intervals of DEGs with sample sizes ranging from 5 to 15 that were randomly selected from 15 pSS and 12 non-pSS. **(E)** Heatmap of the top 100 DEGs. “Red” indicates high relative expression, and “blue” indicates low relative expression.

To better understand the roles of genetic factors in the development of pSS, we investigated the mRNA gene expression profiles of lower labial glands from 27 clinically suspected female pSS patients using RNA sequencing. Among these patients, 15 met the 2016 ACR/ EULAR classification criteria or 2012 ACR classification criteria for pSS with an average age of 49 years, while 12 with the dryness symptoms of mouth or eye with an average age of 49.08 years did not meet these criteria, and were defined as non-pSS (Table 1). In addition, in all pSS patients, we found that there were 10 (66.67%) clinic indexes in high IgG levels, 14 (93.33%) with focus score ≥ 1, 12 (80%) in anti-SSA positivity, 12 (80%) in anti-SSB positivity and 15 (100%) in ANA positivity (Table 1). On the contrary, there were only 2 (16.67%) clinic indexes in high IgG levels, 1 (8.33%) with focus score ≥ 1, 5 (41.67%) in anti-SSA positivity and 2 (16.67%) in anti-SSB positivity in all non-pSS (Table 1). An average of 23.73Gb of clean data (79,091,289 reads) per sample was generated, of which 88.69% could be mapped to the human reference genome. We subsequently assessed the absolute expression level of each gene based on the reads per kilobase of transcript per million mapped reads (RPKM) in DESeq2 (23) package with transcript annotations from GENCODE v74. PCA analysis of gene expression profiles revealed that pSS samples and non-pSS were clearly separated into two distinct clusters with obvious spatial separation (Figure 1A), indicating that substantial gene expression changes are involved in the pathogenesis of pSS.

Using an FDR threshold of 0.05 and an FC threshold of 2, 293 genes were detected to be differentially expressed in pSS vs. non-pSS. Interestingly, we found that the majority (285/293) of DEGs were up-regulated and only 8 genes were down-regulated (Figure 1B and Supplementary Table 2). The top 20 DEGs between pSS and non-pSS are given in Supplementary Table 3. Among them, several previously reported genes were identified, which have previously been demonstrated to participate in the pathogenesis of pSS, including *IRF1, CXCL9* (IFN-gamma), *CXCL10, MS4A1*(*CD20*) (24), *IGHG4* (25), *CXCL13* (26, 27), *TAP1* (28, 29), *CD52* (30), *PSMB8-AS1* (29), *FPR3* (30), *PSMB9* (31), and *GBP1* (32, 33), and some previously unreported DEGs like *IGLV3-19, IGHV1-18, IGHV4-39, TLR10*, and *TYMP*.

To further confirm the robustness of deep sequencing based on expression analysis, a batch of 22 candidate genes were validated by qPCR measurements. A strong correlation [Pearson's correlation (*R*) = 0.88, *P* = 9.49 × 10^−8^] was revealed between the RNA sequencing data and the qPCR results, confirming the consistency of the results derived from the two methods (Figure 1C). To determine whether the marked excess of up-regulated genes compared with down-regulated genes was related to sample size and statistical power, we randomly sampled various sample sizes of the pSS and non-pSS. This demonstrated a steady and remarkable larger quantity of up-regulated genes than down-regulated genes regardless of the sample size (Figure 1D).

### Activation of the Immune System in pSS

**Figure 2:**
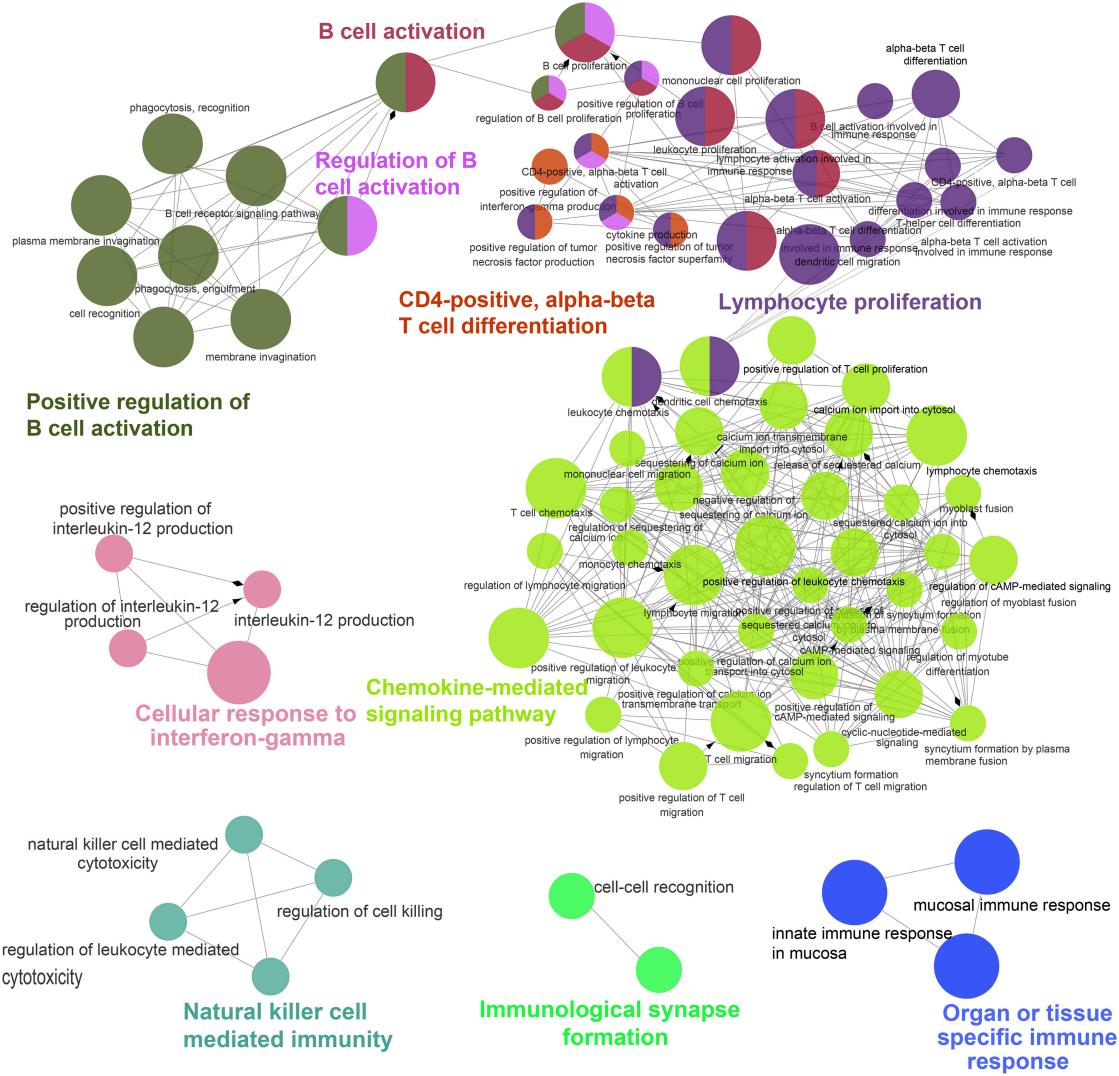
ClueGO network analysis of the top 100 DEGs. Enrichment for GO groups was performed using the ClueGO plugin. The Nodes are colored according to the groups of related functions by statistically significant association of related GO terms. In the groups, only the most significant term is labeled, and the node size corresponds to the significance of each GO term.

To further facilitate interpretation of the biological processes associated with the gene signature in the top 100 DEGs (Figure 1E), the ClueGO (34) plugin for Cytoscape (21) was used to cluster GO terms that participate in the same biological function and to visualize the interactions within and between clusters. As expected, related interconnected networks displayed enrichment of 35 up-regulated DEGs in several groups related to differentiation and activation of immunocytes, which are crucial participants in the process of autoimmunity (Figure 2, Supplementary Figure 1 and Supplementary Table 4), including B cell activation (corrected *P* = 4.4 × 10^−15^), positive regulation of B cell activation (corrected *P* = 8.8 × 10^−12^), regulation of B cell activation (corrected *P* = 5.6 × 10^−13^), lymphocyte proliferation (corrected *P* = 5.7 × 10^−14^), positive T cell selection (corrected *P* = 3.1 × 10^−3^), and CD4-positive, alpha-beta T cell differentiation (corrected *P* = 4.6 × 10^−4^). Furthermore, we found that 21 down-regulated DEGs were significantly enriched in both cellular responses to IFN-gamma (corrected *P* = 1.9 × 10^−4^) and chemokine-mediated signaling pathways (corrected *P* = 2.5 × 10^−6^; Figure 2 and Supplementary Table 5). In addition, functional enrichment was also observed in natural killer cell mediated immunity (GO: 0002228;) and natural killer cell-mediated immunity (GO: 0002228; Figure 2 and Supplementary Table 5).

To evaluate alterations in biological pathways in pSS, the proportions of up-regulated genes in KEGG pathways were calculated (Supplementary Table 6). Several pathways exhibited significant up-regulation patterns in pSS, including systemic lupus erythematosus (hsa05322), alcoholism (hsa05034), viral carcinogenesis (hsa05203), the chemokine signaling pathway (hsa04062), cytokine-cytokine receptor interaction (hsa04060), cell adhesion molecules (CAMs) (hsa04514), Th17 cell differentiation (hsa04659), and herpes simplex infection (hsa05168). In addition, significant alterations were seen in other immunologically-relevant KEGG sub-pathways including primary immunodeficiency (hsa05340), the intestinal immune network for IgA production (hsa04672), Th1 and Th2 cell differentiation (hsa04658), antigen processing and presentation (hsa04612), and natural killer cell mediated cytotoxicity (hsa04650). We found that 34 and 8 DEGs were associated with systemic lupus erythematosus and rheumatoid arthritis KEGG pathways, respectively.

### Close Interactions Among DEGs Associated With Four Enriched Groups in pSS

Despite the prevalence of gene up-regulation in the development of pSS, we also detected 8 down-regulated genes exceeding the FDR threshold of 0.05 and the FC threshold of 2, including *CAMP, FZD10, ITLN1, C2CD4A, CSN3, GH1, CNMD*, and *BPIFB1*. Among these genes, *CAMP* and *FZD10* were the most down-regulated, with FCs of 4.93 and 4.74, respectively (Supplementary Table 2). In enrichment analysis, the organ or tissue specific immune response group (corrected *P* = 7.3 × 10^−5^) was significantly down-regulated in pSS compared to non-pSS (Figure 2 and Supplementary Table 5). Two downregulated genes (*BPIFB1* and *CAMP*) in this group had been reported to be involved in interstitial lung disease, psoriasis, rosacea, or other skin inflammatory disorders (35, 36). Although these both genes were rarely reported to be associated with pSS, a study on experimental dry eye mouse model found *CRAMP*, the ortholog of human *CAMP*, was down regulated for mRNA and protein in the dry eye mouse (37). In addition, the down-regulated DEGs were not significantly enriched in any KEGG pathway. Based on extensive literature research, the relationship between these downregulated genes and pSS has rarely been documented, unlike the relationship between the up-regulated genes and pSS.

**Figure 3:**
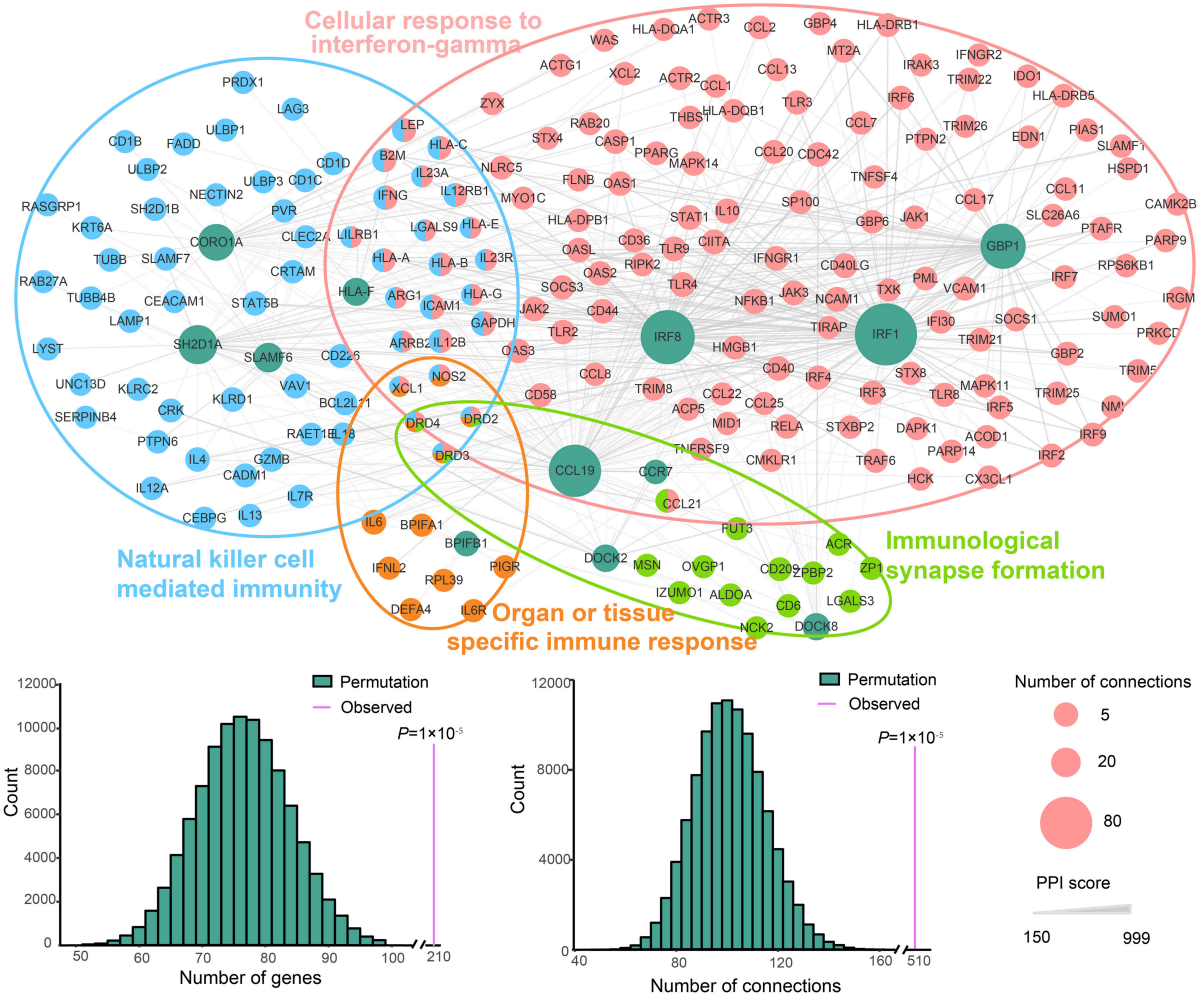
PPI network of the DEGs enriched in natural killer cell-mediated immunity, cellular response to IFN-gamma and organ or tissue specific immune response pathways. The nodes represent genes. Green indicates the DEGs involved in the three pathways, and the other colors correspond to the other genes in these pathways. The size of nodes indicates the number of connections. The edges denote the interactions between two genes, and the width of an edge denotes the score of a genetic interaction. A permutation test of the network genes and connections was performed with 100,000 iterations.

All DEGs involved in four groups, including three up-regulated GO groups (natural killer cell mediated immunity, cellular response to IFN-gamma and immunological synapse formation) and one down-regulated GO group (organ or tissue specific immune response), and the other genes in these groups were used to perform a PPI network analysis. An interconnected network using a comprehensive human protein interactive dataset collected from the STRING database was generated (Figure 3). To prove that the constructed PPI and co-expression networks were not random, we employed a permutation test with 100,000 iterations. Our evaluation of the PPI network showed statistical significance for the number of interacting proteins (*P* = 1 × 10^−5^) and connections (*P* = 1 × 10^−5^), indicating these DEGs have close interaction relative to random expectations, at a protein level. Among the interconnected networks, 11 candidate genes including *CCL19, IRF8, IRF1, GBP1, HLA-F, SH2D1A, CORO1A, SLAMF6*, and *BPIFB1*, were found to be highly likely to have direct interactions with 209 other genes associated with these groups.

### Enhanced Immunological Synapse Formation in pSS

**Figure 4:**
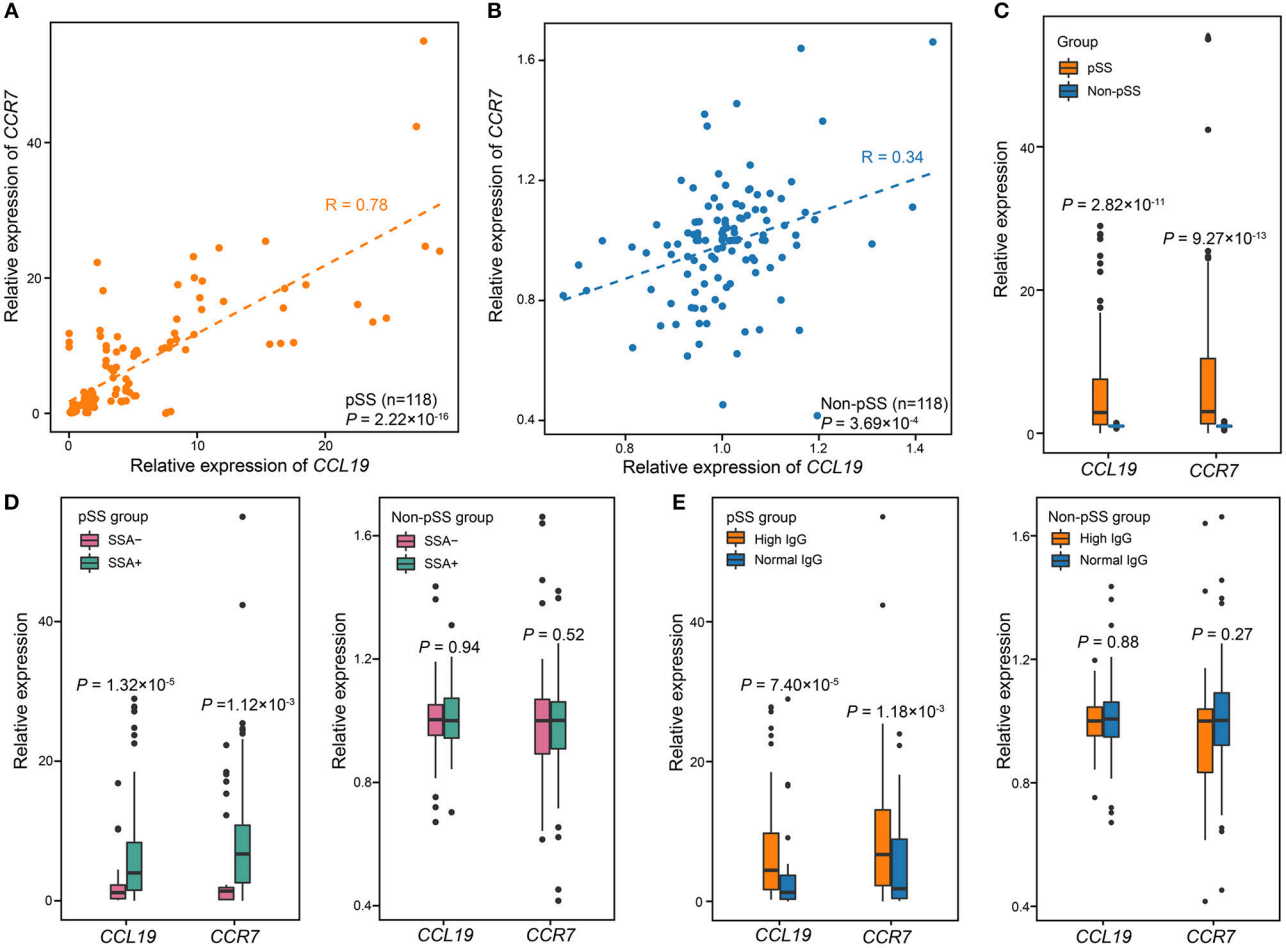
The expression levels of *CCL19* and *CCR7* are associated with pSS. **(A)** Correlation between the expression of *CCL19* and that of *CCR7* in pSS (*n* = 118). The expression of *CCL19* was highly corrected with expression of *CCR7* (Pearson's test, *R* = 0.85, *P* = 2.22 × 10^−14^). **(B)** Correlation between the expression of *CCL19* and *CCR7* in non-pSS (*n* = 118). The two were positively correlated (Pearson's test, *R* = 0.34, *P* = 3.69 × 10^−4^). **(C)** The validation of gene expression of *CCL19* and *CCR7* using qPCR in 118 pSS and 118 non-pSS. **(D)** The relative expression of *CCL19* and *CCR7* was significantly different comparing SSA+ and SSA-cases. **(E)** Comparisons of the relative gene expression of *CCL19* and *CCR7* and serum IgG level in the case and non-pSS groups. *P*-values were obtained from two-sample *t*-test.

According to the biological function analysis of DEGs between pSS patients and non-pSS, the up-regulated genes in pSS showed significant enrichment in immunological synapse formation. In the enriched group, there were four up-regulated genes including *CCL19, CCR7, DOCK2*, and *DOCK8*. Moreover, we also found that both *CCL19* and *CCR7* also belong to cellular response to IFN-gamma group associated with pSS and *CCL19* exhibited a strong connection with members in this group. To further demonstrate the role of immunological synapse formation in the disease process of pSS, the gene expression of key molecules involved in immunological synapse formation, such as *CCL19* and *CCR7*, was further validated by qPCR in a larger cohort consisting of 118 pSS patients and 118 non-pSS (Supplementary Table 1). We found that gene expression of *CCL19* and *CCR7* were positively correlated with each other in pSS patients (*R* = 0.78, *P* = 2.22 × 10^−16^; Figure 4A) and non-pSS (*R* = 0.34, *P* = 3.69 × 10^−4^; Figure 4B). Both *CCL19* and *CCR7* expression were significantly increased in patients compared with that in non-pSS (*P* = 2.82 × 10^−11^ for *CCL19, P* = 9.27 × 10^−13^ for *CCR7*; Figure 4C).

The association of these genes with clinical manifestations was also analyzed in these 118 pSS patients and 118 non-pSS. We separated pSS cases and non-pSS into anti-SSA-positive and anti-SSA-negative groups, respectively, and found that the expression levels of both *CCL19* and *CCR7* were higher in anti-SSA-positive pSS than anti-SSA-negative pSS (*P* = 1.32 × 10^−5^ for *CCL19, P* = 1.12 × 10^−3^ for *CCR7*; Figure 4D). In contrast, no significant difference was found between anti-SSA positive and anti-SSA negative non-pSS. In addition, we separated pSS cases and non-pSS into high and normal serum IgG groups, respectively, and found that the expression levels of both *CCL19* and *CCR7* were significantly correlated with high serum IgG levels in pSS (*P* = 7.40 × 10^−5^ for *CCL19, P* = 1.18 × 10^−3^ for *CCR7*; Figure 4E), while no significant correlation was found in non-pSS. For focus score, qPCR validation showed the expression of *CCR7*/*CCL19* in pSS cases with focus score ≥1 was higher than that in pSS cases with focus score <1, although this difference was not statistically significant (*P* = 0.054 for *CCL19, P* = 0.2 for *CCR7*; Supplementary Figure 2A). In addition, there was no difference for the expression of both genes between non-pSS samples with focus score ≥1 and that with focus score <1 (*P* = 0.73 for *CCL19, P* = 0.47 for *CCR7*; Supplementary Figure 2B). We also recruited healthy volunteers without sicca symptoms and found that there was no difference between the *CCR7*/*CCL19* expression of healthy controls and non-pSS group (*P* = 0.93 for *CCL19, P* = 0.55 for *CCR7*; Supplementary Figure 3).

### Up-Regulated Gene Expression Can Serve as an Index to Predict pSS

**Figure 5:**
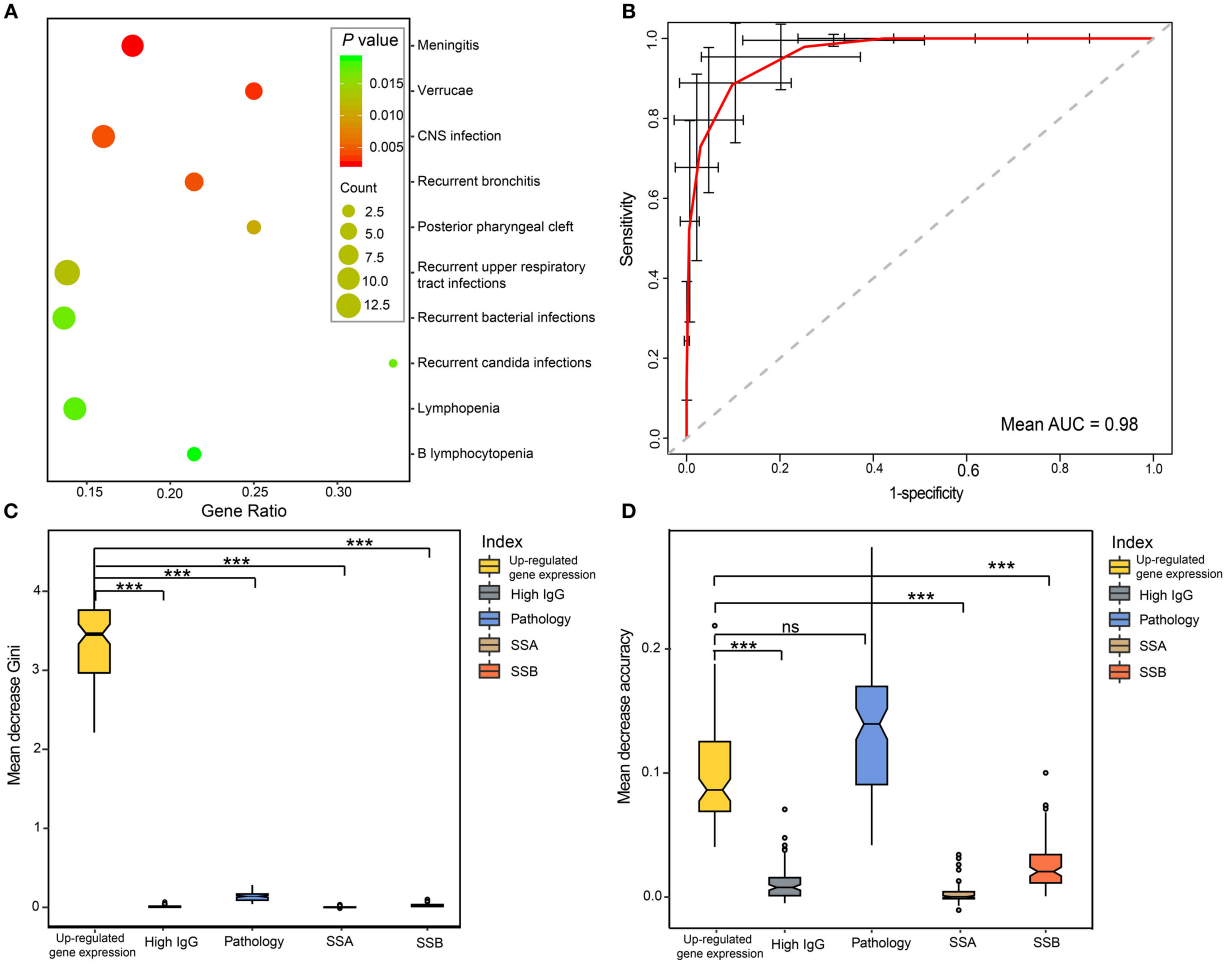
Performance of DEGs in the diagnosis of pSS. **(A)** Top10 phenotype enrichment based on the DEGs in pSS. The scatterplots display the enriched phenotypes, and the sizes of the dots indicate the number of genes. **(B)** ROC curve of pSS prediction for the RF model using CVs. The red curve was the average of 100 randomized cross-validation runs, with error bar showing standard deviation. The mean AUC of the 100 runs was 0.98. **(C,D)** Variable importance measures of clinical variables in predicting pSS. The mean decrease accuracy indicates the decrease in model accuracy when a variable is excluded, and the mean decrease Gini measure indicates the discriminant capabilities of a variable during the construction of the model. Differences between CVs were analyzed by ANOVA test, and Nemenyi test was used for multiple comparisons. “^***^” represented the adjusted *P* < 0.001. ns, no significance; RF, random forest; CV, clinical variables.

To further assess the role of DEGs in pSS, phenotypic terms of enrichment for all DEGs were generated based on the HPO database. We found that 21 phenotypes were significantly enriched that were related to immunity, infection and cancer (all *P* < 0.05; Figure 5A and Supplementary Table 7). Meningitis and verrucae were the most highly enriched (*P* = 2.4 × 10^−3^), followed by central nervous system (CNS) infection, recurrent bronchitis, recurrent upper respiratory tract infections, recurrent bacterial infections, and recurrent candida infections. In addition, clinical presentations such as lymphopenia (38), autoimmune thrombocytopenia (39), and vasculitis (40), are common in pSS, and were also found in our cohort (Supplementary Table 7). All these results showed an important role of the DEGs in the development of pSS, thus, these DEGs can serve as potential biomarkers for the diagnosis of pSS.

To evaluate the performance of DEGs in the diagnosis of pSS, receiver operating characteristic (ROC) curve analysis was performed based on up-regulated gene expression and typical features of pSS, including pathology, SSA positivity, SSB positivity and hypergammaglobulinemia. Up-regulated gene expression showed a high discriminatory accuracy in identifying pSS, with a high mean AUC value of 0.98 (Figure 5B). To further assess the contribution of each variable to the overall diagnostic performance in pSS, random forests (RFs) were performed using ranking methods. We found that up-regulated gene expression was the most important index for predictive capability among all of the features in mean decrease Gini (Figure 5C). Moreover, the discriminant capability of the model was greatly reduced if the up-regulated gene expression was excluded from the model. Although the mean decrease accuracy of up-regulated gene expression was slightly smaller than that of positive pathology of the salivary gland, the up-regulated gene expression identified in our cohort were more discriminatory than other clinic features, based on RF modeling (Figure 5D).

## Discussion

In this study, we applied RNA-seq to compare gene expression profiles in well-established pSS patients to those of non-pSS. We ultimately identified several key pathways that are dysregulated in pSS compared to non-pSS, and showed that gene expression changes performed well in discriminating pSS cases. We found that, in comparison with non-pSS, pSS exhibited a distinct gene expression pattern primarily composed of up-regulated genes. Among them, there were a number of well-documented genes, such as *IRF1* (41), *CXCL9* (IFN-gamma) (33, 42, 43), and *CXCL10* (44), that are associated with IFN signaling and are known to participate in the pathogenesis of pSS by inducing the perpetuation of inflammation autoantibody production, and glandular cell apoptosis (13, 15, 33, 43, 45). Additionally, we also found several up-regulated KEGG pathways which suggest a potential role for microorganism in the disease process.

In addition to IFN signaling, GO interpretation of DEGs revealed enrichment in the differentiation and activation of immunocytes and in chemokine-mediated signaling pathways. Chemokines/chemokine receptors are essential for maintaining the function and interaction of T lymphocytes (46). In this study, we observed several highly overexpressed chemokines/chemokine receptors in pSS, including *CCL18, CCR1, CXCL11, CXCL13*, and *CXCR4*, and the IFN signaling-associated chemokines mentioned above (like *IRF1, CXCL9*, and *CXCL10*). KEGG pathway analysis also revealed the overactivity of both B-cell and Natural killer cell, and significant alterations in Th1, Th2, and Th17 cell differentiation; chemokine signaling pathway; cytokine-cytokine receptor interactions, CAMs; and antigen processing and presentation, further supporting overactivation of the immune system in pSS.

The immunological synapses, which were originally named after neuronal synapses, represent the interface between antigen-presenting cells or target cells and lymphocytes (47), and are necessary for T cell signaling and differentiation (48, 49). For the first time, we found that DEGs in pSS were enriched in immunological synapse formation, and the key molecules in this pathway, such as the cytokine *CCL19* and its chemokine receptor *CCR7*, were up-regulated in pSS. By binding to *CCR7, CCL19* attracts certain cells of the immune system, including dendritic cells (50), antigen-engaged B cells (51), and CCR7^+^ central-memory T-Cells (52). It has been reported that the *CCL19*/*CCR7* chemokine system is expressed in inflamed muscles of polymyositis and inclusion body myositis (IBM) and may be involved in the pathogenesis of polymyositis (53) and IBM (54). In patients with rheumatoid arthritis, *CCL19* may reflect blood B cell disturbances and predict clinical responses to rituximab (RTX) (55). The up-regulation of *CCL19* was also observed in patients with primary and secondary SS compared to another autoimmune disorder or control, and was considered to contribute to the leukocyte microenvironmental homing in MSG from SS patients (56). Likewise, the expression levels of *CCL19* are significantly elevated in both hip ligament tissue and serum of ankylosing spondylitis (AS) patients compared to normal controls, suggesting a role of *CCL19* in AS pathogenesis (57). Recently, an increased frequency of CCR7^+^CD4^+^ T cells, which was shown to be closely correlated with EULAR Sjögren's syndrome disease activity index (ESSDAI), was found in pSS patients, suggesting that *CCR7* might participate in the development of pSS by mediating the migration of CD4^+^ cells (58). In addition, *CCR7* was also found to be expressed highly in CD34+ cells attracted to MIP-3β as well as in the input cord blood CD34+ cells, indicating that CCR7 might function on migration of activated T cells to inflamed tissue (59). In light of these observations, we further investigated the gene expression levels of *CCL19*/*CCR7* in the salivary glands, which are characterized by lymphocytes infiltration in pSS. *CCL19*/*CCR7* expression was found to be up-regulated in the salivary glands of pSS patients compared to non-pSS. Furthermore, the gene expression levels of *CCL19*/*CCR7* were positively correlated with anti-SSA antibody and IgG levels in pSS patients, indicating an overproduction of autoantibodies during the development of pSS, which might be promoted by enhanced immunological synapse.

Among the top up-regulated genes found in the salivary gland of pSS patients in this study, most have been identified in the parotid gland saliva or peripheral blood of primary SS patients in previously published studies, such as *MS4A1*(*CD20*) (24), *IGHG4* (25), *CXCL13* (26, 27), *TAP1* (28, 29), *CD52* (30), *PSMB8-AS1* (29), *FPR3* (30), *PSMB9* (31), and *GBP1* (32, 33). However, we found that some previously unreported DEGs with potential role in etiopathogenesis of pSS. For example, genes encoding the V region of the variable domain of immunoglobulin heavy or light chains, which participates in antigen recognition, were also up-regulated; these genes included *IGLV3-19* (60), *IGHV1-18* and *IGHV4-39*. *TLR10*, as a modulatory pattern-recognition receptor without known ligand specificity or biological function, was also found to be elevated in pSS. Another up-regulated gene, *TYMP*, encodes an angiogenic factor that promotes angiogenesis *in vivo* and stimulates the *in vitro* growth of a variety of endothelial cells. Mutations in this gene have been associated with mitochondrial neurogastrointestinal encephalomyopathy (61). These genes provide a foundation for further study of the pathogenesis of pSS.

Previous genome-wide association studies have revealed shared risk polymorphisms among systemic lupus erythematosus, rheumatoid arthritis and pSS (62, 63). In this study, we likewise found that there was significant enrichment of DEGs shared between pSS and these two immunologically mediated diseases, indicating shared pathways of these three autoimmune diseases. The results of our analysis using a ranking method with an RF model showed that up-regulated gene expression was the most important index for the diagnosis of pSS, with greater discriminator capacity than other clinical, immunological and even histopathological features of pSS. Up-regulated gene expression even showed better diagnostic performance than pathological positivity of the salivary gland in mean decrease Gini, which is a hallmark of pSS and has been placed, due to its great importance, in the American-European Group Consensus (AEGC) criteria (9) and the classification criteria proposed by the ACR in 2012 (10). Overall, our results indicate that the up-regulated genes between pSS patients and non-pSS can be used to diagnose pSS with a high mean AUC value of 0.98. Therefore, these DEGs may serve as attractive targets for the development of clinically useful biomarkers.

One limitation of this study is the small sample size used for RNA-seq of pSS patients, although to ensure robustness of positive findings, 118 additional pSS patients and 118 non-pSS have been recruited for validation of candidate gene expression. Although the present sample size is capable of detecting most of the DEGs, further larger studies are needed to fully characterize the expression changes associated with pSS. Furthermore, many of the DEGs found in this study need to be further investigated to determine their biological function and likely pathogenic role in pSS. This study also only investigated mRNA, and thus the landscape of other RNA types, including circular RNA and non-coding RNAs remain the subject of future studies.

In conclusion, we observed a high prevalence of up-regulated genes in the development of pSS that promote the activation of the immune system in this disease. Significantly elevated *CCL19*/*CCR7* expression in the salivary gland in pSS, which was correlated with anti-SSA antibody positivity and elevated IgG levels in pSS, implicates enhanced immunological synapses in the disease process and suggests their contribution to lymphocyte activation and autoantibody production in the pSS process. The contrasting gene expression pattern demonstrated between pSS and non-pSS deepens our understanding of disease mechanisms and suggests that these DEGs are a promising index for the detection of pSS.

## Ethics Statement

This study was approved by the Ethics Committee of the First Affiliated Hospital of Wenzhou Medical University, and written informed consent was received from all participants for their enrollment.

## Author Contributions

ZL, FL, and AP contributed to the drafting and revision of the manuscript, data acquisition, and analysis. CZ, HX, and SJ contributed to experimentation, data analysis, and manuscript revision. MJ, JF, and XZ contributed to patient recruitment, data acquisition, and manuscript revision. MB and XW contributed to study concept and design, critical review, and manuscript revision. All authors read and approved the final manuscript.

### Conflict of Interest Statement

The authors declare that the research was conducted in the absence of any commercial or financial relationships that could be construed as a potential conflict of interest.

## Supplementary Material

Supplementary Figure 1 Enrichment for GO groups in adaptive immune response, leukocyte migration and positive T cell selection for the top 100 DEGs.

Supplementary Figure 2 Expression level of *CCL19* and *CCR7* in different population grouped by focus score. **(A)** The expression of *CCL19* and *CCR7* in pSS with focus score ≥ 1 and in pSS with focus score < 1 (*P* = 0.054 for *CCL19, P* = 0.2 for *CCR7*; two-sample *t*-test) **(B)** The expression of *CCL19* and *CCR7* in non-pSS with focus score ≥ 1 and in non-pSS with focus score < 1(*P* = 0.73 for *CCL19, P* = 0.47 for *CCR7;* two-sample *t*-test).

Supplementary Figure 3 The *CCR7*/*CCL19* expression in healthy controls and non-pSS (*P* = 0.93 for *CCL19, P* = 0.55 for *CCR7*; two-sample *t*-test).

Supplementary Table 1 Clinical informations of pSS and non-pSS used for qPCR.

Supplementary Table 2 Differentially expressed genes between the pSS patients and non-pSS.

Supplementary Table 3 Top 20 DEGs between the pSS patients and non-pSS.

Supplementary Table 4 The ClueGo terms of the top100 up-regulated genes.

Supplementary Table 5 The ClueGo terms of down-regulated genes.

Supplementary Table 6 The KEGG terms of differentially expressed genes.

Supplementary Table 7 Phenotype enrichment based on DEGs.
