# Supplementary figures and images for: Elevated CCL19/CCR7 Expression During the Disease Process of Primary Sjögren's Syndrome
Source: Front Immunol. 2019 Apr 24;10:795. doi: 10.3389/fimmu.2019.00795 (PMC6491632; doi:10.3389/fimmu.2019.00795)

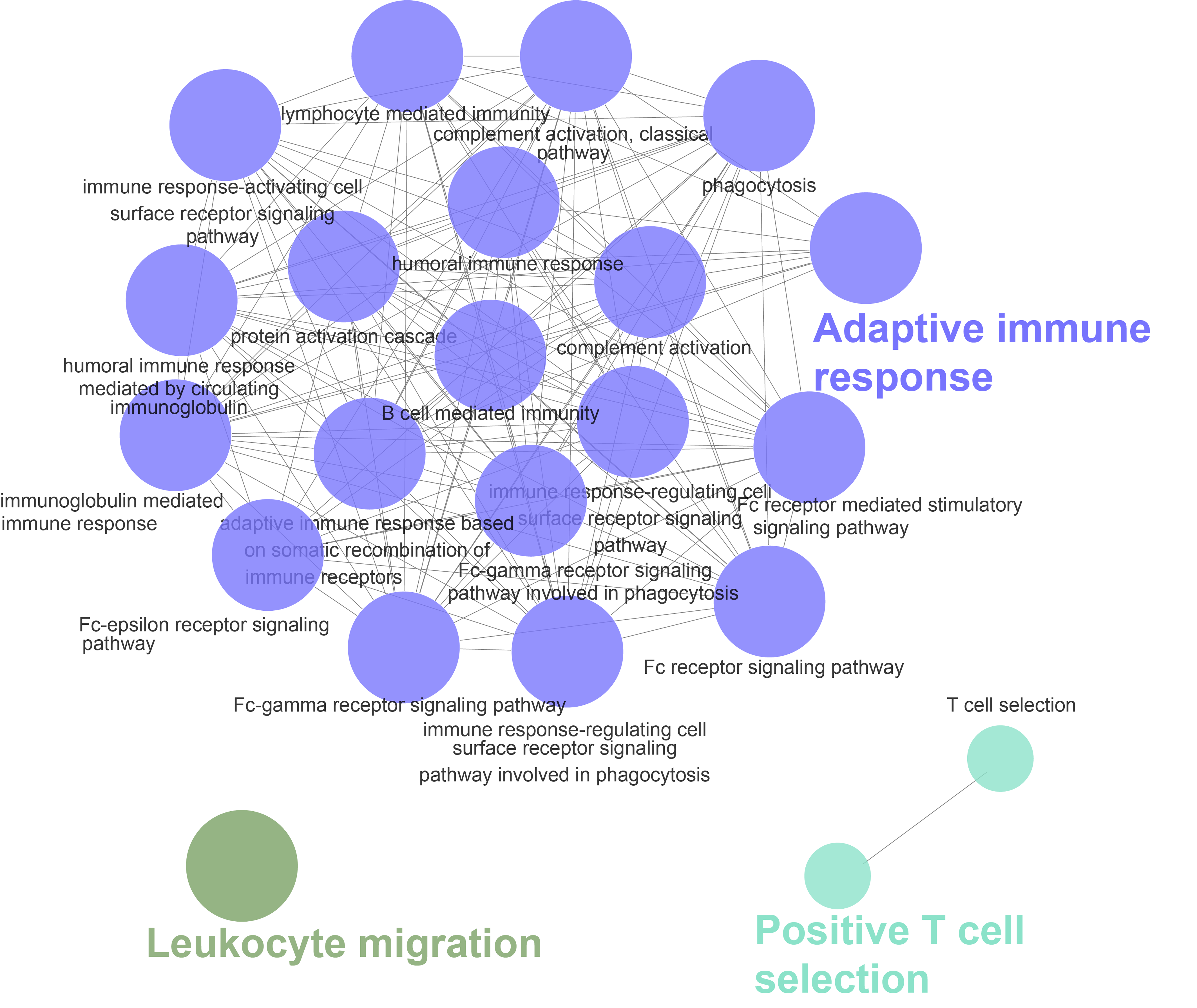

Supplement: Supplementary Figure 1 — Enrichment for GO groups in adaptive immune response, leukocyte migration and positive T cell selection for the top 100 DEGs. [file Image_1.TIF]

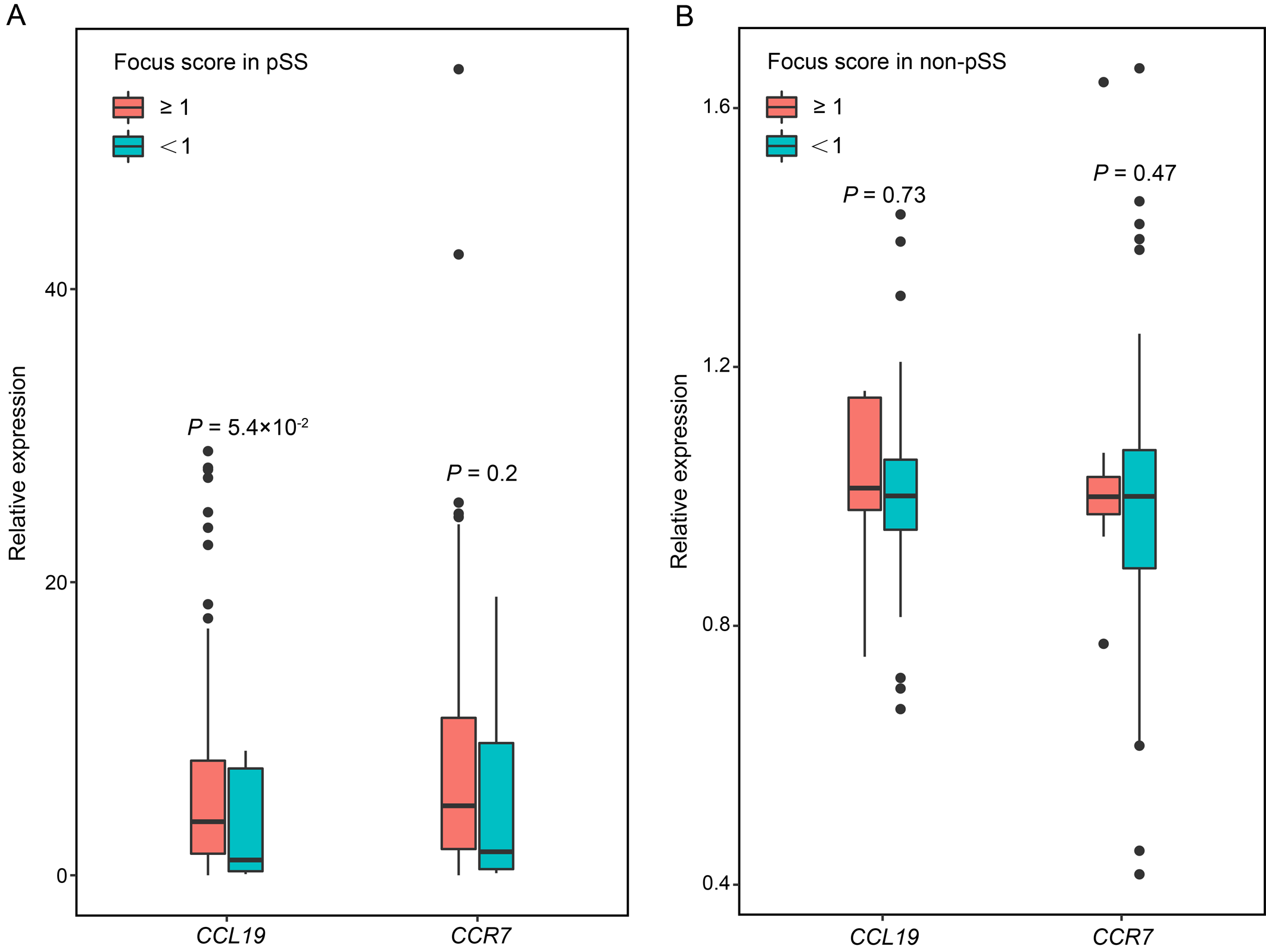

Supplement: Supplementary Figure 2 — Expression level of CCL19 and CCR7 in different population grouped by focus score. (A) The expression of CCL19 and CCR7 in pSS with focus score ≥ 1 and in pSS with focus score < 1 (P = 0.054 for CCL19, P = 0.2 for CCR7; two-sample t-test) (B) The expression of CCL19 and CCR7 in non-pSS with focus score ≥ 1 and in non-pSS with focus score < 1(P = 0.73 for CCL19, P = 0.47 for CCR7; two-sample t-test). [file Image_2.TIF]

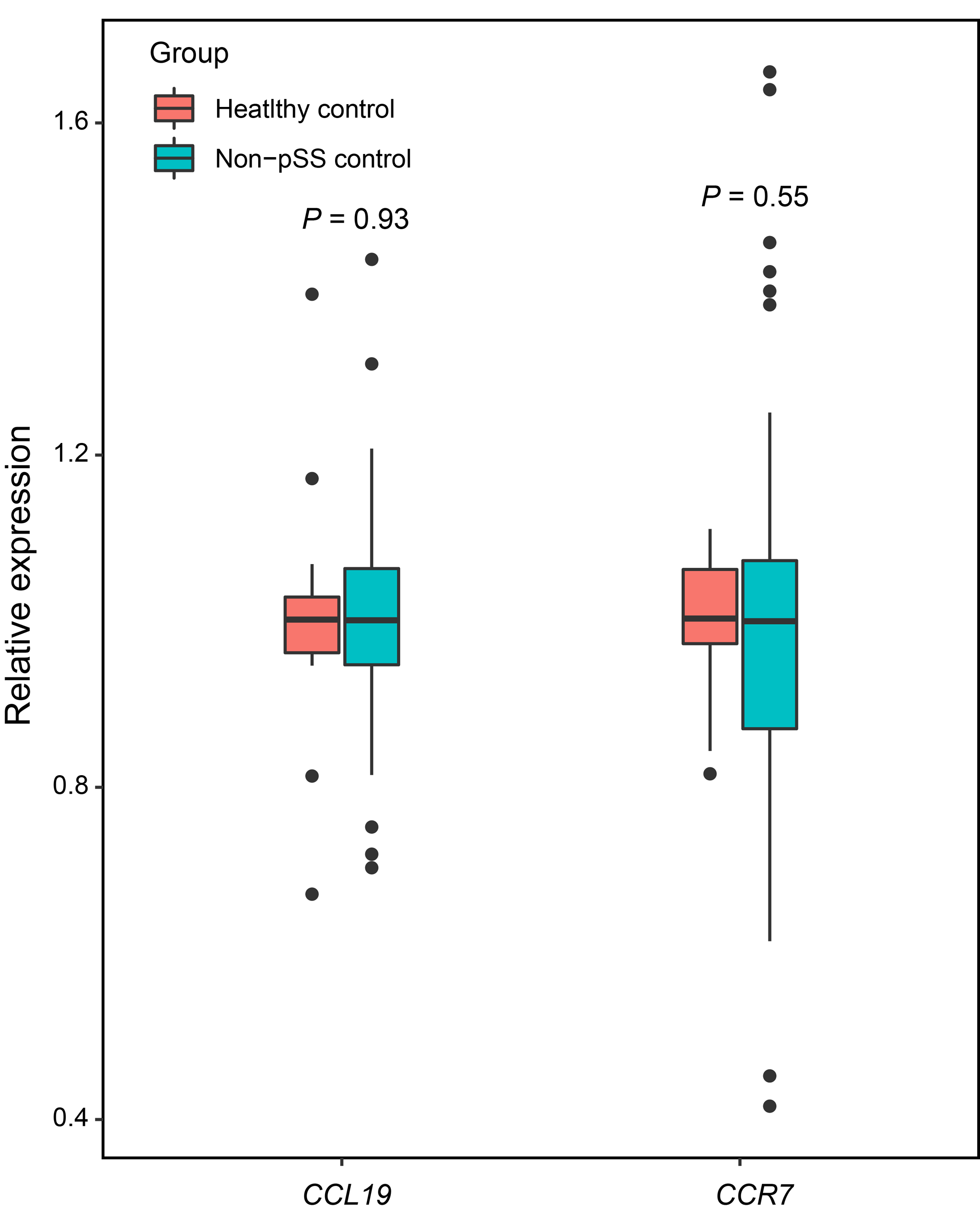

Supplement: Supplementary Figure 3 — The CCR7/CCL19 expression in healthy controls and non-pSS (P = 0.93 for CCL19, P = 0.55 for CCR7; two-sample t-test). [file Image_3.TIF]
