# Supplementary material for: Elevated CCL19/CCR7 Expression During the Disease Process of Primary Sjögren's Syndrome
Source: Front Immunol. 2019 Apr 24;10:795. doi: 10.3389/fimmu.2019.00795 (PMC6491632; doi:10.3389/fimmu.2019.00795)
Supplement: Supplementary Table 3 — Top 20 DEGs between the pSS patients and non-pSS. [file Table_3.DOCX]

**Supplementary TABLE 3 |** Top 20 DEGs between the pSS and non-pSS.

| **Gene Symbol** | **Description** | **Log_2_ FC** | ***P* Value** | **FDR** | **Type** |
| --- | --- | --- | --- | --- | --- |
| *CXCL9* | C-X-C Motif Chemokine Ligand 9 | 2.58 | 2.72×10^-16^ | 6.10×10^-12^ | Up |
| *MS4A1* | Membrane Spanning 4-Domains A1 | 3.46 | 7.09×10^-14^ | 7.95×10^-10^ | Up |
| *CXCL10* | C-X-C Motif Chemokine Ligand 10 | 2.50 | 1.23×10^-13^ | 9.16×10^-10^ | Up |
| *IGHG4* | Immunoglobulin Heavy Constant Gamma 4 | 3.37 | 2.52×10^-12^ | 1.30×10^-8^ | Up |
| *CCL19* | C-C Motif Chemokine Ligand 19 | 2.42 | 2.89×10^-12^ | 1.30×10^-8^ | Up |
| *CXCL13* | C-X-C Motif Chemokine Ligand 13 | 3.85 | 3.48×10^-12^ | 1.30×10^-8^ | Up |
| *TAP1* | Transporter 1, ATP Binding Cassette Subfamily B Member | 1.29 | 4.18×10^-12^ | 1.34×10^-8^ | Up |
| *IGHV1-18* | Immunoglobulin Heavy Variable 1-18 | 3.17 | 4.79×10^-12^ | 1.34×10^-8^ | Up |
| *PSMB8-AS1* | PSMB8 Antisense RNA 1 | 1.24 | 4.44×10^-11^ | 1.11×10^-7^ | Up |
| *TYMP* | Thymidine Phosphorylase | 1.36 | 7.21×10^-11^ | 1.62×10^-7^ | Up |
| *OR2I1P* | Olfactory Receptor Family 2 Subfamily I Member 1 Pseudogene | 2.15 | 8.67×10^-11^ | 1.66×10^-7^ | Up |
| *CD52* | CD52 Molecule | 2.30 | 8.9×10^-11^ | 1.66×10^-7^ | Up |
| *IGLV3-19* | Immunoglobulin Lambda Variable 3-19 | 2.82 | 1.02×10^-10^ | 1.76×10^-7^ | Up |
| *FPR3* | Formyl Peptide Receptor 3 | 1.35 | 1.14×10^-10^ | 1.82×10^-7^ | Up |
| *PSMB9* | Proteasome Subunit Beta 9 | 1.46 | 2.17×10^-10^ | 3.25×10^-7^ | Up |
| *IRF1* | Interferon Regulatory Factor 1 | 1.10 | 3.78×10^-10^ | 5.30×10^-7^ | Up |
| *GBP1* | Guanylate Binding Protein 1 | 1.15 | 6.30×10^-10^ | 8.31×10^-7^ | Up |
| *TLR10* | Toll Like Receptor 10 | 2.16 | 8.06×10^-10^ | 1.00×10^-6^ | Up |
| *IGHV4-39* | Immunoglobulin Heavy Variable 4-39 | 2.30 | 1.34×10^-10^ | 1.58×10^-6^ | Up |
| *C16orf54* | Chromosome 16 Open Reading Frame 54 | 1.08 | 1.83×10^-10^ | 2.05×10^-6^ | Up |
